# Adjustable Method for Real-Time Gait Pattern Detection Based on Ground Reaction Forces Using Force Sensitive Resistors and Statistical Analysis of Constant False Alarm Rate

**DOI:** 10.3390/s18113764

**Published:** 2018-11-03

**Authors:** Fangli Yu, Jianbin Zheng, Lie Yu, Rui Zhang, Hailin He, Zhenbo Zhu, Yuanpeng Zhang

**Affiliations:** 1 School of Information Engineering, Wuhan University of Technology, Wuhan 430070, China; yufangli_aewa@163.com (F.Y.); zhengjb@whut.edu.cn (J.Z.); zhangr@whut.edu.cn (R.Z.); windy@whut.edu.cn (H.H.); 2 School of Electronic and Electrical Engineering, Wuhan Textile University, Hongshan District, Wuhan 430073, China; 3 Hubei Key Laboratory of Biomass Fibers and Eco-dyeing & Finishing, Wuhan Textile University, Wuhan 430200, China; 4 AirForce Early Warning Academy, Wuhan 430000, China; zzbradar@126.com (Z.Z.); zhangyuanpeng312@163.com (Y.Z.)

**Keywords:** ground contact forces, force sensitive resistors, threshold method, constant false detection probability method

## Abstract

A new approach is proposed to detect the real-time gait patterns adaptively through measuring the ground contact forces (GCFs) by force sensitive resistors (FSRs). Published threshold-based methods detect the gait patterns by means of setting a fixed threshold to divide the GCFs into on-ground and off-ground statuses. However, the threshold-based methods in the literature are neither an adaptive nor a real-time approach. To overcome these drawbacks, this study utilized the constant false alarm rate (CFAR) to analyze the characteristics of GCF signals. Specifically, a sliding window detector is built to record the lasting time of the curvature of the GCF signals and one complete gait cycle could be divided into three areas, such as continuous ascending area, continuous descending area and unstable area. Then, the GCF values in the unstable area are used to compute a threshold through the CFAR. Finally, the new gait pattern detection rules are proposed which include the results of the sliding window detector and the division results through the computed threshold. To verify this idea, a data acquisition board is designed to collect the GCF data from able-bodied subjects. Meanwhile, in order to test the reliability of the proposed method, five threshold-based methods in the literature are introduced as reference methods and the reliability is validated by comparing the detection results of the proposed method with those of the reference methods. Experimental results indicated that the proposed method could be used for real-time gait pattern detection, detect the gait patterns adaptively and obtain high reliabilities compared with the reference methods.

## 1. Introduction

Walking is the basic capability of human being to move from one gait cycle to another which allows people to carry out their daily lives [1]. One complete gait cycle in human walking consists of two main gait patterns, such as stance phase and swing phase. Specifically, stance phase is defined when the foot is in contact with the ground, while swing phase is defined when the foot totally leaves the ground [2]. Gait analysis is a useful means to quantify the state of the gait patterns, which can be accomplished by a great number of sensor platforms, including force sensors [3,4], inertial sensors [5,6,7,8], air pressure sensors [1], inclinometer sensors [9], foot switches [10] and electromyography (EMG) sensors [11,12].

Among all the sensor platforms, force sensor platforms, such as force sensitive resistors (FSRs), can represent the gold standard method for gait analysis [13]. FSRs can be mounted in shoe soles to measure the ground contact forces (GCFs). The electrical resistance change of an FSR is proportional to the GCF induced by human foot. As reported in Reference [1], each gait pattern has a unique GCF pattern such that the measuring value changes of FSRs can be directly correlated to the gait patterns.

Generally, the division of gait patterns in force sensor platform is based on the threshold method which sets a threshold to divide the GCF into on-ground and off-ground statuses. As described by Smith et al. [14], 80% of the detection errors using FSRs was due to the setting of threshold value such that many researchers presented their approaches to compute an appropriate threshold. Mariani et al. [4] defined the threshold as 5% body weight with the result that the weight of each subject should be measured before the experiments. Lopez-Meyer et al. [15] and Catalfamo et al. [16] took use of the maximum and minimum GCFs of gait cycles to calculate the threshold, which meant that the GCF should be post-processed after data acquisition. However, Lie Yu et al. [17] and Jing Tang et al. [18] declared that the methods in Reference [4,15,16] were not adaptable to different people and different walking speeds. On one hand, for Mariani method [4], different subjects were usually with different body weights such that different thresholds should be computed for different subjects. Meanwhile, no matter at what speed the subject walked, one constant threshold was set for the same subject in all experiments. On the other hand, the Lopez-Meyer method [15] and Catalfamo method [16] could not be used for real-time gait pattern detection because the maximum and minimum GCFs were obtained in data post-processing. Therefore, Lie Yu et al. proposed the proportional method (PM) which calculated the sums and proportions of GCFs for gait pattern detection. Two proportional factors were used for all subjects in all experiments and this PM achieved high average reliability. Jing Tang et al. [18] presented the self-tuning triple-threshold algorithm (STTTA) which could search out the maximum and minimum GCFs in real time. Three initial threshold values were set for all subjects in all experiments and the three thresholds would be adjusted to adapt the human walking. However, there existed an obvious drawback for PM that one parameter in Reference [17] was determined by the attachment between the shoe and the foot, which would cut down the detection accuracy of the whole system. Additionally, the proposed STTTA in Reference [18] used three thresholds to calculate a new threshold for the future walking gait cycle. Nevertheless, the calculation formulas were extremely simple which only relied on the maximum and minimum GCFs in the current walking gait cycle.

In this paper, the constant false alarm rate (CFAR) is utilized to analyze the characteristic of the GCF signals. Specifically, a sliding window detector is built to record the lasting time of the curvature of the GCF signals and one complete gait cycle could be divided into three areas, such as continuous ascending area, continuous descending area and unstable area. Then, the GCF values in the unstable area are used to compute a threshold through the CFAR. Finally, the new gait pattern detection rules are proposed which include the results of the sliding window detector and the division results through the computed threshold. In order to test the detection reliability of the proposed method, five previous approaches [4,15,16,17,18] in the literature were chosen as reference methods to obtain comparative results.

The aim of this study is to develop a real-time method to detect the gait patterns adaptively. Specifically, this proposed method is irrelevant to subjects’ body weights and walking speeds. To evaluate the availability and reliability of the proposed method, five published method are introduced as references. The detection results of the proposed method are compared with the reference methods.

## 2. Method

### 2.1. Subjects

This study included twelve males and ten females of average age 23.1 ± 3.2 years and average mass 68.2 ± 7.6 kg with no history of foot diseases or limitations. Before the experiment, the subjects gave their written informed consent for participation in this study as the purpose was explained in detail to each of them and their safeties could be ensured. These subjects were selected from the postgraduate students of Wuhan University of Technology, which approved our research.

### 2.2. Measurement Principle of FSR Sensors

**Figure 1:**
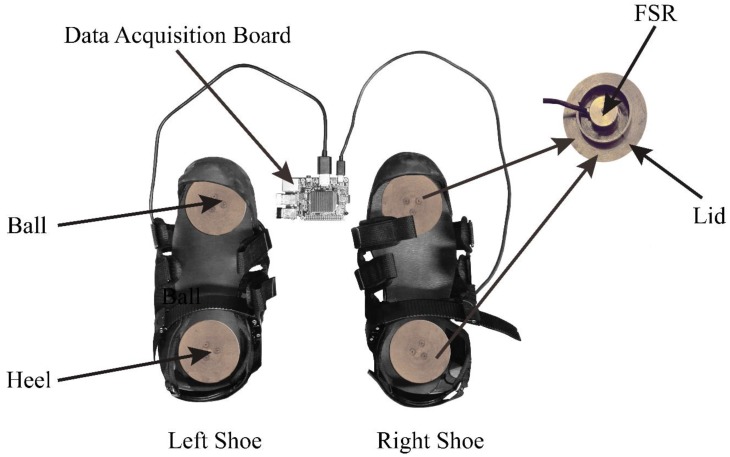
FSRs placed inside each shoe in the ball and in the heel and a data acquisition board is used to collect the GCF signal.

To validate our research, we designed a gait phase detection system as shown in Figure 1. Two sets of FSRs (LOSON LSH-10, LOSON Instrumentation, Nankin, China) were severally embedded into the insole of the ball and heel of each shoe. The FSRs signal were digitalized by a high resolution of 16 bits AD converters at a sample frequency of 1000 Hz. Each FSR possesses a wide measuring range of 0–200 kg with a high accuracy (including linearity and repeatability) of ± 0.5% full scale (FS). As the FSR sensor outputs a weak micro-voltage-level signal, the output signal should be amplified to a voltage-level signal. Meanwhile, a pressure tester (TLS-S1000W, Jinan Zhongchuang Industry Test System Co., Ltd., Jinan, China) is used to calibrate the FSRs such that the amplified output signal within a range of 0–5 V correlates with the measured mass of 0–200 kg.

After data collection, the acquired FSR signals were filtered by Butterworth low pass filter with a cut-off frequency of 200 Hz to eliminate the unnecessary high frequency noise.

### 2.3. Description of Walking Experiments

After sensor calibrations, the experiments were implemented to test the reliability of CFAR for gait pattern detection. Then, each subject was asked to perform 5 trials to wear the designed shoes to walk. The five trials were performed on treadmill for 30 s per trial at a designated constant speed of 2 km/h, 3 km/h, 4 km/h, 5 km/h and 6 km/h in turn. 

### 2.4. Gait Pattern Detection Algorithm

For human walking, a complete gait cycle could be divided into two main phases such as stance-phase and swing-phase. Stance-phase means that the foot is in contact with the ground, while swing-phase means that the foot is off the ground. As two FSR sensors are mounted severally inside the heel and ball of shoe, it would result in that only one point (i.e., the ball or the heel) is contacting the ground and the other point is off the ground. As a result, single point contact could lead to the transition detection between the two phases. To be specific, the transition from swing-phase to stance-phase is Heel-Strike, while the transition from stance-phase to swing-phase is Heel-Off. To differ from the two transitions, stance-phase is renamed as Full-Stance (i.e., two points are in contact). To distinguish these gait patterns, the proposed algorithm is presented in the following.

#### 2.4.1. Statistical Characteristic Analysis

**Figure 2:**
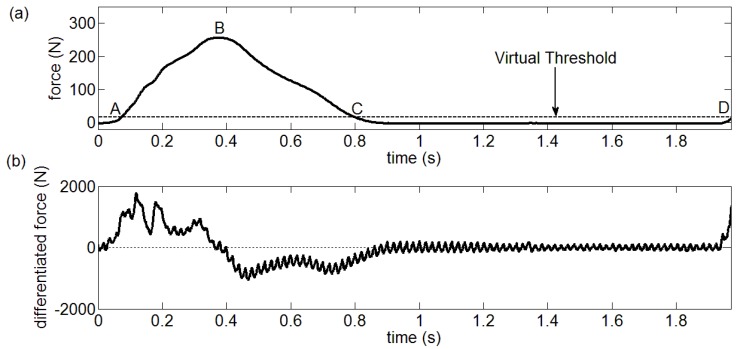
(**a**) Using virtual threshold to divide the GCF into on-ground and off-ground statuses, (**b**) The differential GCFs are used to identify the References [A B], [B C] and [C D].

The gait pattern detection mainly focuses on the division of on-ground and off-ground statuses through setting a threshold. As a result, this paper carries out the characteristic analysis of the two statuses. Firstly, a virtual threshold is set to divide the GCFs into on-ground and off-ground statuses. Figure 2a demonstrates the division made by the virtual threshold in a complete gait cycle. The maximum value in this complete cycle divides the GCFs distinguished as on-ground status into two intervals, including [A B] and [B C] as shown in Figure 2a. Figure 2b depicts the difference of GCFs. The curvature in Reference [A B] interval is basically positive, which lasts a long time. Meanwhile, the curvature in Reference [B C] interval is mainly negative, which also lasts a long time. However, the curvature polarity (i.e., positive or negative) in Reference [C D] interval is unstable and the determined curvature polarity would change when lasting a short time.

Based on these reasons, a sliding window detector could be built to record the lasting time of the curvature of the GCF signals. Given two types of sliding windows, such as ascending window and descending window, which can be described as

(1) $$\left\{ \begin{array}{l} A_{W}(1:N_{A})=[0,0,\cdots ,0]\\ D_{W}(1:N_{D})=[0,0,\cdots ,0] \end{array}\right.\;$$

where *A_w_* is the ascending window function and *N_A_* is the size of *A_W_*. Meanwhile, *D_W_* is the descending window function and *N_D_* is the size of *D_W_*.

When the present point (i.e., noted as *F*) of GCF signals comes, its derivative is computed and noted as *dF*. When the *dF* value is positive, the ascending window would be sliding but the descending window remains unchanged, which could be written as

(2) $$\left\{ \begin{array}{l} A_{W}(1:N_{A}-1)=A_{W}(2:N_{A})\\ A_{W}(N_{A})=1\\ D_{W}(1:N_{D}-1)=D_{W}(2:N_{D})\\ D_{W}(N_{D})=0 \end{array}\right.\;$$

On the other hand, when the *dF* value is negative, the descending window would be sliding but the ascending window remains unchanged.

(3) $$\left\{ \begin{array}{l} A_{W}(1:N_{A}-1)=A_{W}(2:N_{A})\\ A_{W}(N_{A})=0\\ D_{W}(1:N_{D}-1)=D_{W}(2:N_{D})\\ D_{W}(N_{D})=1 \end{array}\right.\;$$

Then, the numbers of “1” in the two window are counted, which are separately noted as *C_A_* and *C_D_*. *C_A_* is the number of “1” in the ascending window, while *C_D_* is for descending window.

**Figure 3:**
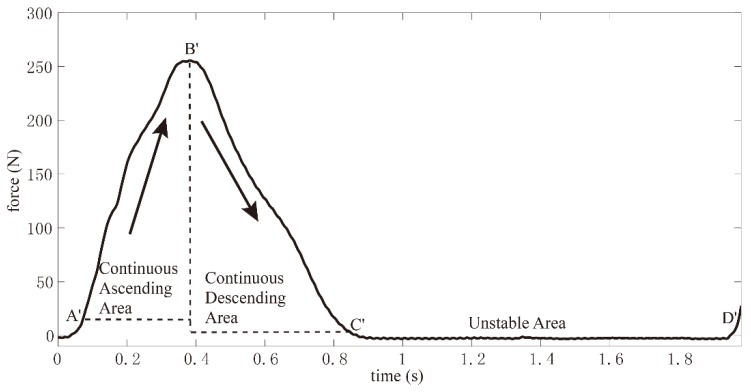
Sliding window detector divides the GCF into three parts.

Finally, count limits *L_A_* and *L_D_* are set for *C_A_* and *C_D_*, respectively. When *C_A_* (or *C_D_*) is larger than *L_A_* (or *L_D_*), it can be considered that the curvature lasts a long time. Additionally, when the *C_A_* is smaller than *L_A_* and the *C_D_* is smaller than *L_D_*, it can be considered that the curvature lasts a short time. When proper values are chosen for these parameters, the GCFs signals can be processed in the Figure 3. However, the [A’ B’] and [B’ C’] intervals could not be considered as on-ground status. As a result, this statistical characteristic analysis would lead to false detection such that further research should be carried out.

#### 2.4.2. Constant False Alarm Rate and Threshold Computation

For all of the relating studies, the set threshold is closer to the GCFs differentiated as off-ground status. Therefore, threshold computation should be made after [C’ D’] interval is searched using sliding window detector. The error between GCF value at point D’ and the computed threshold would lead to false detection. As a result, CFAR is used to make up this drawback.

It is assumed that *H*_0_ is the detection result of GCF identified as true off-ground status and *H*_1_ is the detection result of GCF identified as true on-ground status. Meanwhile, *D*_0_ represents that the GCF is detected as off-ground status, while *D*_1_ represents that the GCF is detected as on-ground status. In this paper, the threshold value is calculated based on the analysis of [C’ D’] interval which can be distinguished as off-ground status. Therefore, it would happen that the division results obtained by the computed threshold are detected as on-ground statuses, while the true detection results are off-ground statuses. Then, the false detection probability in this case can be expressed as

(4) $$P_{F}=P(D_{1}|H_{0})=\int_{T}^{\infty }p(x|H_{0})dx\;$$

where *P_F_* is the false detection probability and *P*(*D*_1_|*H*_0_) is the probability that judges *H*_0_ as *D*_1_. Meanwhile, *x* is the GCFs happening in the [C’ D’] interval, *T* is the detection threshold and *P*(*x*|*H*_0_) is the probability density function.

**Figure 4:**
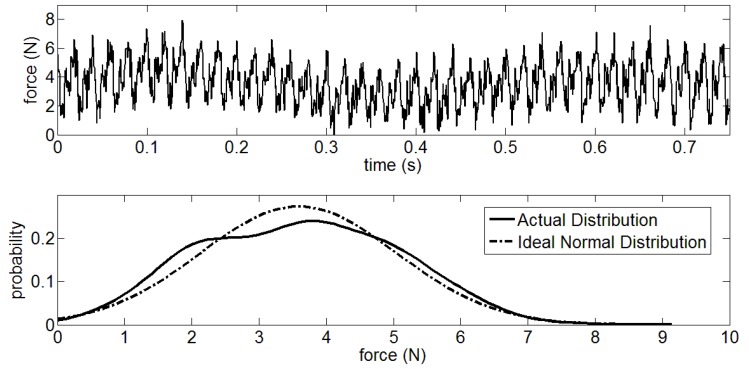
Data analysis of normal distribution for GCF in unstable area.

**Figure 5:**
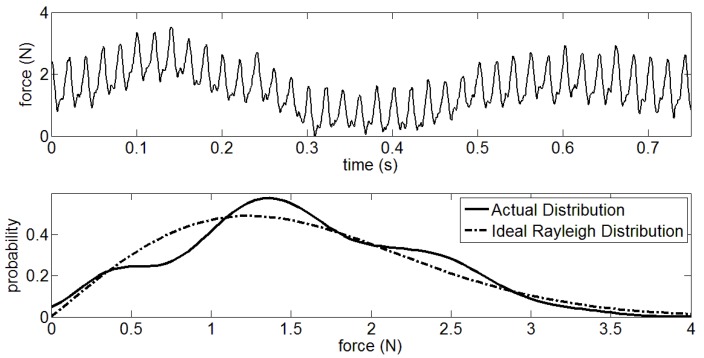
Data analysis of Rayleigh distribution for GCF in unstable area.

As shown in Figure 4, the unfiltered *x* in the [C’ D’] interval is subject to normal distribution. However, the filtered *x* obeys the Rayleigh distribution as pictured in Figure 5. Therefore, the *P*(*x*|*H*_0_) can be described as

(5) $$p(x|H_{0})=\frac{x}{\sigma^{2}}e^{\left(-\frac{x^{2}}{2\sigma^{2}}\right)}\;$$

where *σ* the signal density of *x*. Substituting the Equation (5) into Equation (4), the false detection probability can be rewritten as

(6) $$P_{F}=\int_{T}^{\infty }\frac{x}{\sigma^{2}}e^{\left(-\frac{x^{2}}{2\sigma^{2}}\right)}dx=e^{\left(-\frac{T^{2}}{2\sigma^{2}}\right)}\;$$

Then, the detection threshold *T* can be obtained in the following.

(7) $$T=\sqrt{-2\sigma^{2}InP_{F}}\;$$

**Figure 6:**
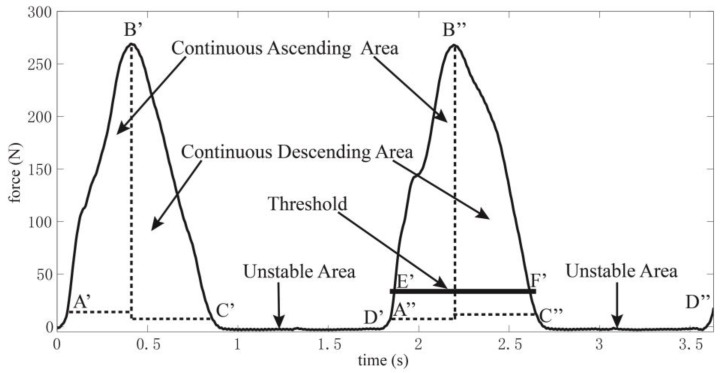
For the first gait cycle, the sliding window detector divides GCFs into continuous ascending area, continuous descending area and unstable area. The GCFs in the unstable area from the first gait cycle are used to compute a threshold for the next gait cycle according to the CFAR.

**Figure 7:**
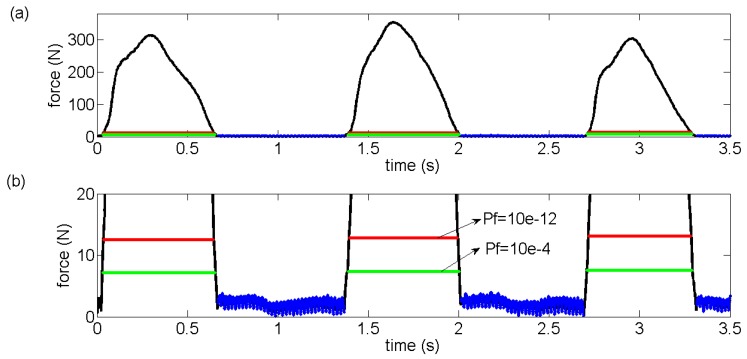
Using different *P_F_* gains for threshold computation. The line marked in blue is the GCFs detected to be in unstable area. The lines marked in red and green are the computed threshold using two different *P_F_* values. (**a**) The original signal analysis, (**b**) The exaggerating signal analysis for better demonstration.

When the [C’ D’] interval is searched through the sliding window detector, the detection threshold would be computed immediately at point D.’ In Rayleigh distribution, *σ* is proportional to the average of *x*, which can be expressed as

(8) $$\sigma =\sqrt{\frac{2}{\pi }}\bar{x}\;$$

where *x* is the average value of *x* in Reference [C’ D’] interval. The GCF values after D’ point would be compared to the detection threshold *T* to divide the GCFs into on-ground and off-ground statuses. As shown in Figure 6, [A’’ B’’] and [B’’ C’’] are the continuous ascending and descending areas for the next gait cycle, respectively. Meanwhile, A’’ and D’ are the same point. In point A’’ (or D’), a threshold is computed according to Equation (7). Then, the status division can be made, which can be stated as follows.

(9) $$S=\left\{ \begin{array}{l} 1,\;F\geq T\\ 0,\;F<T \end{array}\right.\;$$

where *F* is the GCF after D’ (or A’’) point. For *S*, “1” indicates an on-ground status and “0” indicates an off-ground status. As depicted in Figure 6, this calculated threshold divides the next gait cycle with the result that two intersection points E’ and F’ are obtained. Based on the division formula in Equation (9), [A’’ E’] and [F’ C’’] intervals are both judged as off-ground status. However, the *P_F_* value would affect the threshold value according to Equation (7), which could be exactly described in Figure 7. The GCFs marked in blue are detected to be in unstable area. The lines marked in red and green are the computed threshold using two different *P_F_* values. Therefore, the gait patterns could be distinguished according to the rules in Table 1, when both the status divisions of ball and heel are made.

### 2.5. Evaluation of the Results

In order to test the reliability of the proposed method, reference methods should be determined. As well known, the Lopez-Meyer method had been compared with the “GAITRite system” and acquired a comparative and reliable confidence of 95% [15]. Therefore, not only the Lopez-Meyer method [15] but also the other methods [4,16,17,18] in the literature were introduced as reference methods. The gait pattern detection rules of these methods were severally presented in Reference [17,18]. Hence, the reliability of this study is determined by comparing the detection results between the reference methods and the proposed algorithm. Finally, the obtained reliabilities were processed to measure the "test-retest reliability" by taking several measurements on each subject. The analysis is performed with the intraclass correlation coefficient (ICC) proposed by Bartko [19].

## 3. Results

### 3.1. Selection of Coefficients

**Figure 8:**
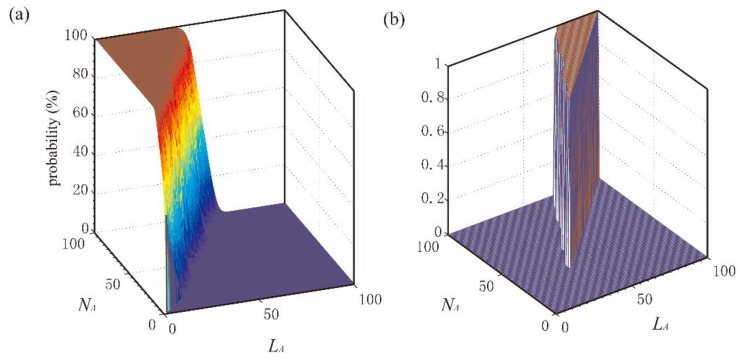
Parameter optimum for ascending window. (**a**) Using traversing search method to optimize *L_A_* and *N_A_* for ascending window, (**b**) Search the optimal *L_A_* and *N_A_* when testing probability is zero.

**Figure 9:**
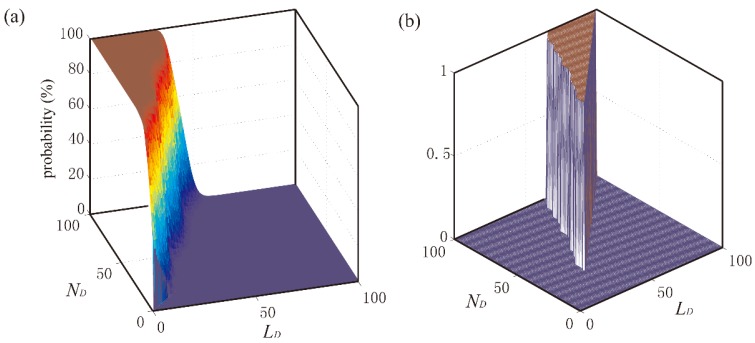
Parameter optimum for descending window. (**a**) Using traversing search method to optimize *L_D_* and *N_D_* for descending window, (**b**) Search the optimal *L_D_* and *N_D_* when testing probability is zero.

Before the experiments, the sizes (i.e., *N_A_* and *N_D_*) and lengths (i.e., *L_A_* and *L_D_*) of the ascending and descending windows should be optimized. Firstly, one million of the GCF points judged as off-ground status through the reference methods were used to test the probability that the GCF points failed to be divided into unstable area. Secondly, the traversing search method was used to optimize these parameters, which are shown in Figure 8a and Figure 9a. The testing probability is smaller, the parameter selection is better. When the testing probability is zero, a great many of parameter combinations could be figured out. On the other side, the optimization algorithm should obey two conditions that *L_A_* is smaller than *N_A_* and *L_D_* is smaller than *N_D_*. Therefore, the testing probability results of being zero are extracted and described in Figure 8b and Figure 9b. Bigger value of these parameters would lead to the lag to detection results such that the smallest parameter values in Figure 8b and Figure 9b is chosen. As a result, optimum values of *L_A_* = 35, *N_A_* = 38, *L_D_*= 36 and *N_D_* = 40 were used.

**Figure 10:**
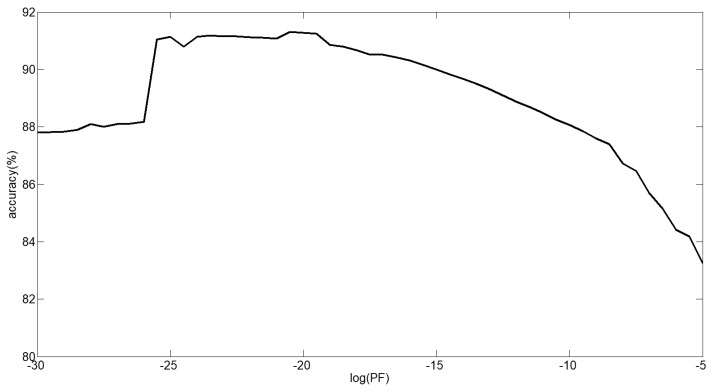
Parameter optimum for *P_F_*.

To determine the highest reliability of the proposed algorithm, *P_F_* were optimized using the traversing search method. Choosing the data from 5 subjects as training data, optimum values of *P_F_* = 20.5 were used, which is shown in Figure 10.

### 3.2. Results of Gait Pattern Detection

As shown in Table 2, the proposed algorithm was highly reliable when compared with the five reference methods. The average reliabilities were 90.15%, 89.83%, 89.45%, 89.98% and 88.90% when compared with the TAM [16], Lopez-Meyer [15], PM [17], STTTA [18] and Mariani method [4], respectively. The average ICC of reliabilities was 0.56, which proved to be fair according to the guidelines of Cicchetti [20] and moderate according to the guidelines of Koo and Li [21].

**Figure 11:**
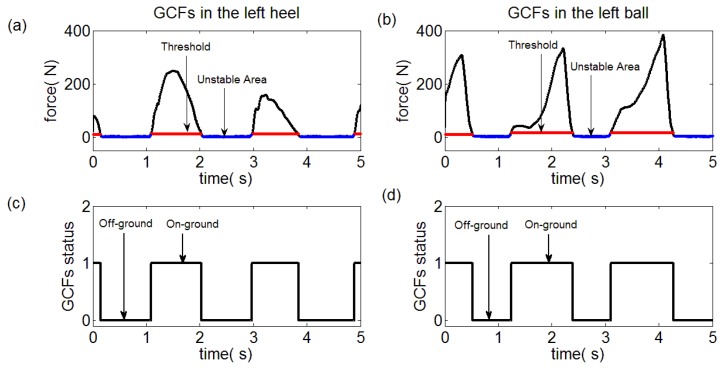
Status division for left ball and heel. (**a**) GCFs in the left heel, (**b**) GCFs in the left ball, (**c**) Status division for GCFs in the left heel, (**d**) Status division for GCFs in the left ball.

**Figure 12:**
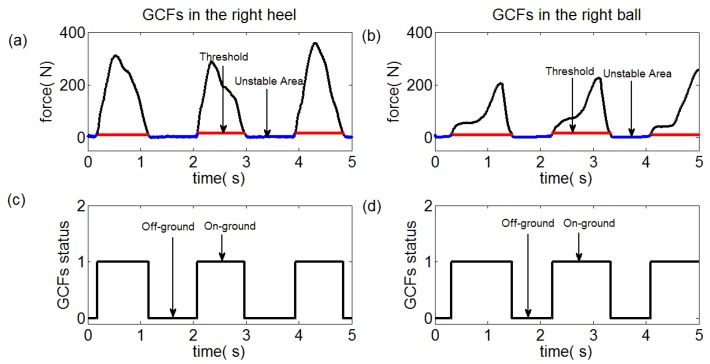
Status division for right ball and heel. (**a**) GCFs in the right heel, (**b**) GCFs in the right ball, (**c**) Status division for GCFs in the right heel, (**d**) Status division for GCFs in the right ball.

**Figure 13:**
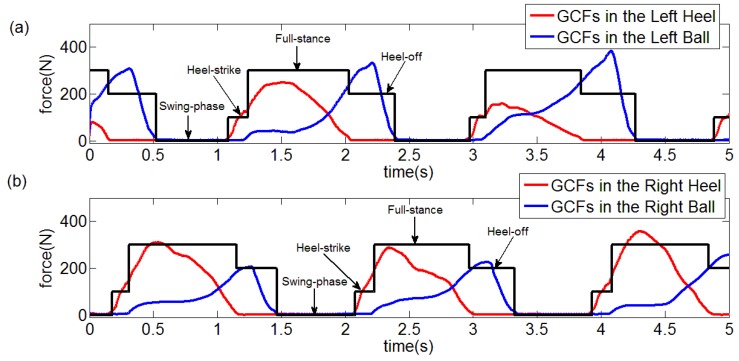
(**a**) Results of gait pattern detection for left foot, (**b**) Results of gait pattern detection for right foot.

In this paper, the collected GCF data were processed through the proposed algorithm. The GCFs in the left ball and heel were severally pictured in Figure 11a, b along with the unstable area and computed threshold marked. Then, the GCFs status division was made through the Equation (9), which was depicted in Figure 11c,d. Similarly, the processing results of the right foot were demonstrated in Figure 12. It was qualitatively illustrated in Figure 13 where the gait patterns were identified according to the rules in Table 1.

On one side, the detection results of the proposed method were depicted in Figure 11, Figure 12 and Figure 13. On the other side, the detection results of the reference methods were not provided due to the high reliabilities which would lead to high similarity comparing with Figure 11, Figure 12 and Figure 13. 

### 3.3. Real-Time Application for Gait Pattern Detection

In this gait pattern detection system, the sampling frequency was set to be 1000 Hz. After data acquisition in each time, the data processing, including the sliding window detector, threshold computation based on CFAR, status division and gait pattern detection, could be actualized within one sampling period. In fact, the time delay between data acquisition and detection result in one running cycle was less than 1 ms. As a result, the proposed algorithm can be used for gait pattern detection in real time.

## 4. Discussion

### 4.1. Advantages of the Research

When compared with the five reference methods, the proposed method is highly reliable (as seen in Table 2) and can identify the gait patterns in real time. The proposed method can be adaptable to different subjects with different body weights at different walking speeds, because adaptive thresholds are calculated according to the GCF signal characteristic analyzed though the sliding window detector and the CFAR. As reported in Reference [17], the attachment between the shoe and foot would lead to bigger change of GCF magnitude in the unstable area such that the PM reliability would be affected to be lower. However, this situation would not happen in this proposed method as the lasting time of the differential GCF is used by the sliding window detector to identify the GCF in the unstable area.

The STTTA [18] and the proposed method can both achieve to adaptively identify the gait patterns in real time. However, the STTTA can identify the gait patterns using the GCFs data in the current period. Meanwhile, the proposed method applied the sliding window detector using the GCFs data in a lasting time which consisted of 35 (or 36) sampling periods. Specifically, for STTTA, when the GCFs in the heel or ball are judged to be off-ground status, a sudden change of GCF signal would lead to the GCF value to be larger than the threshold and judged to be on-ground status. Therefore, misdetection would happen for STTTA in this situation. Nevertheless, this situation will not happen in this proposed method, because the gait pattern is distinguished and given from the analysis of detection results in 35 (or 36) sampling periods.

According to the detection rules in Table 1, when the GCFs are identified to be in the unstable area, these GCF signals are judged to be off-ground status. When the heel and ball are both judged to be off-ground status, the specific gait pattern is swing phase. In swing phase, if the foot trembles leading to relatively larger GCF values, the sliding window detector would still identify the GCF signals to be in unstable area. Meanwhile, the CFAR algorithm would figure out a larger threshold to avoid the GCF signals identified as on-ground status.

For lower exoskeleton robot systems, the gait pattern detection plays an extremely important role. The results of gait pattern detection correlate with motion intention of the wearer such that the robotic leg automatically move to stand or swing. Meanwhile, the lower exoskeleton robot systems need real-time and adaptive gait pattern detection for different wearers to move at different walking speeds. This proposed method would meet this need.

The detection results were obtained from able-bodied subjects and a cyclic gait pattern sequence could be analyzed for healthy people using this research. However, the gait pattern sequence of hospital patients would differ from that of healthy people. Therefore, this research would help with the diagnosis of some persons with walking injuries, diseases or limitations.

Most studies [5,22,23] used the accelerometers and gyroscopes to obtain the acceleration and velocity for gait pattern detection. Some gait patterns in these studies did not appear in this paper, such as toe-off and initial-contact. If we want to identify the toe-off phase on this force platform, another FSR should be added to be mounted inside the top of the shoe. Meanwhile, a relatively small threshold was set for the GCF in the heel to detect the initial-contact.

### 4.2. Limitation of the Research

When the subject is walking at a faster speed, the lasting time of the GCF signals identified to be in the unstable area would get less such that the data points in this area reduce. In this situation, the data points in this area would not obey Rayleigh distribution strictly. To overcome this drawback, sampling frequency should be guaranteed to be high enough.

As reported in Reference [18], the cut-off frequency of Butterworth low pass filter is 10 Hz. However, the GCF signal should not be filtered excessively in this proposed method, which would destroy the data distribution characteristic.

When the subject jumps, both feet are judged to be swing-phase. In the walking experiments, subject jumping was rarely observed and only occurred when the subjects were running at a sufficiently rapid pace.

## 5. Conclusions

This paper uses the statistical theory to detect the gait patterns in real time. The statistical theory consists of sliding window detector and CFAR algorithm. The sliding window detector divides the GCFs into three areas, such as continuous ascending area, continuous descending area and unstable area. Then, the CFAR algorithm calculated adaptive thresholds according to GCF signals identified to be in the unstable area. The status division would be made according the adaptive threshold and the area division result. Finally, the gait pattern could be distinguished based on the detection rules. Experimental results indicated that the proposed method could be used for real-time gait pattern detection, detect the gait patterns adaptively and obtain high reliabilities compared with the reference methods.

## Figures and Tables

**Table 1:** Rule of gait pattern detection.

Heel		Ball		Gait Pattern
Results of Sliding Window Algorithm	Status Division by Detection Threshold	Results of Sliding Window Algorithm	Status Division by Detection Threshold	
[C’ D’] interval	—	[C’ D’] interval	—	Full-Stance
[A’ C’] interval	1			Heel-Strike
	0			Swing-Phase
[C’ D’] interval	—	[A’ C’] interval	1	Heel-Off
			0	Swing-Phase
[A’ C’] interval	1		1	Full-Stance
	0		0	Swing-Phase

**Table 2:** Reliability of the proposed method compared with the reference methods.

Subject	Gender	Compared with TAM Method	Compared with Lopez-Meyer Method	Compared with PM	Compared with STTTA	Compared with Mariani Method
1	Male	91.49%	92.52%	89.85%	88.91%	86.61%
2	Male	90.89%	92.49%	92.31%	91.23%	89.63%
3	Male	86.33%	89.43%	86.35%	92.42%	91.07%
4	Male	90.26%	91.15%	87.96%	85.56%	88.89%
5	Male	88.68%	90.81%	91.23%	89.98%	89.62%
6	Male	90.96%	85.66%	90.96%	91.32%	90.06%
7	Male	92.01%	91.57%	85.65%	92.04%	90.65%
8	Male	89.46%	91.81%	92.12%	87.99%	86.30%
9	Male	89.17%	86.55%	88.64%	88.27%	87.96%
10	Male	85.32%	91.22%	92.31%	84.42%	87.34%
11	Male	89.27%	94.93%	84.69%	89.81%	86.58%
12	Male	88.38%	91.96%	88.96%	88.84%	89.39%
13	Female	86.22%	89.04%	92.35%	92.29%	90.25%
14	Female	87.61%	85.78%	91.20%	91.70%	89.11%
15	Female	90.53%	89.32%	88.99%	90.52%	85.98%
16	Female	96.41%	88.95%	89.62%	90.59%	87.19%
17	Female	90.12%	89.81%	86.34%	91.23%	88.67%
18	Female	92.11%	89.61%	91.37%	93.12%	90.96%
19	Female	94.22%	91.27%	89.91%	90.06%	91.41%
20	Female	91.75%	86.33%	87.65%	88.93%	89.47%
21	Female	91.91%	86.12%	90.03%	87.32%	88.58%
22	Female	91.49%	92.52%	89.85%	93.05%	90.05%
Average	—	90.15%	89.83%	89.45%	89.98%	88.90%
